# The development and validation of a diagnostic scoring system to differentiate pulmonary tuberculosis from non-tuberculosis pulmonary infections in HIV-infected patients with severe immune suppression

**DOI:** 10.1186/s12879-021-06552-3

**Published:** 2021-08-23

**Authors:** Jing Ouyang, Jing Yuan, Yaling Chen, Yanming Zeng, Vijay Harypursat, Yanqiu Lu, Hui Chen, Yaokai Chen

**Affiliations:** 1 Clinical Research Center, Chongqing Public Health Medical Center, Chongqing, China; 2 Division of Infectious Diseases, Chongqing Public Health Medical Center, Chongqing, China; 3 School of Biomedical Engineering, Capital Medical University, No. 10 Youanmenwai Road, Fengtai, Beijing, 100069 China

**Keywords:** Scoring system, Tuberculosis, Human immunodeficiency virus, Diagnosis

## Abstract

**Background:**

It remains challenging to differentiate tuberculosis (TB) from non-TB pulmonary infections in HIV-infected patients. Herein, we developed a scoring system aimed to rapidly determine the likelihood of TB or non-TB pathology in HIV-infected patients presenting with pulmonary infections.

**Methods:**

We collected and collated data of hospitalized HIV-infected patients with pulmonary infections, followed by univariate and multivariate data analyses to determine risk variables that were significantly different between HIV/TB patients and HIV/non-TB patients. Subsequently, a regression coefficient was calculated for each variable, and a score was assigned to each variable in line with its regression coefficient. The sum of the scores for each variable in our scoring model was used to predict the likelihood of TB or non-TB pulmonary infection in each patient. Finally, we tested the diagnostic accuracy of the scoring system in our retrospective cohort, as well as in a prospective cohort.

**Results:**

A total of 598 HIV-infected patients were enrolled in our retrospective cohort, among whom 288 had TB and 310 had non-TB pulmonary infections. Eight variables, including fever, highest body temperature, erythrocyte sedimentation rate (ESR), cervical lymphadenopathy, hilar and/or mediastinum lymphadenopathy, pulmonary cavitation, pleural effusion, and miliary nodules, were found to be mathematically significantly different via univariate analysis and multivariate logistic regression analysis. After regression coefficient calculation followed by score assignment, a receiver operating characteristic (ROC) curve was plotted, and the area under the curve (AUC) was calculated to be 0.902. When the total score for a patient is > 12, the sensitivity and specificity for TB prediction using our scoring system were 76.4% and 87.7% respectively in the retrospective cohort, and its diagnostic accuracy was 82.7% in the prospective cohort.

**Conclusions:**

Our results demonstrate that our proposed diagnostic scoring system could be helpful in differentiating pulmonary TB from non-TB pulmonary infections in HIV-infected patients.

## Background

Human immunodeficiency virus (HIV) infection and tuberculosis (TB) are two major communicable diseases that threaten human health globally. In 2019, about 10 million people developed TB globally, and among them, 8.2% of these individuals were people living with HIV. Moreover, there were an estimated 1.2 million TB deaths among HIV-negative people in 2019, and an additional 208,000 TB deaths among HIV-positive people [1].

As is well established, HIV-infected patients are susceptible to various opportunistic infections as a consequence of a paucity of adequate cellular immunity, among which TB remains the leading cause of hospitalization and death among people living with HIV [2]. For co-infected individuals, the two pathogens, *Mycobacterium tuberculosis* (MTB) and HIV, pose a dual threat which creates a vicious cycle of immune dysfunction among these individuals. On the one hand, MTB increases HIV viral load and genetic mutation, accelerates CD4 + T-cell depletion, and leads to progressive HIV disease [3, 4]. On the other hand, HIV results in a 2–20 times increased risk of TB above baseline rates, and causes more cases of multi-drug resistant TB [3, 4].

Early establishment of TB diagnosis and effective anti-tuberculosis treatment followed by antiretroviral therapy are key steps for the survival of patients co-infected with HIV and TB. However, early diagnosis of TB remains challenging in HIV/TB co-infected patients with advanced immunodeficiency owing to the following reasons: (1) the clinical features and chest radiographic presentations of these patients are often atypical; (2) sputum smear-negative TB is common in those with advanced immunodeficiency and noncavitary disease [5]; (3) sputum culture is time-consuming and may be inaccessible in many resource-limited regions.

In this study, we aimed to establish a scoring system for the diagnosis of TB in HIV-infected patients with advanced immunodeficiency, and evaluate its overall predictive accuracy.

## Materials and methods

### Study method

This study included retrospective and prospective cohorts. The selection criteria for our retrospective cohort were as follows: (1) HIV-infected patients admitted to the Division of Infectious Diseases of Chongqing Public Health Medical Center; (2) Diagnosis of HIV/AIDS was consistent with Chinese Guidelines for Diagnosis and Treatment of HIV/AIDS [6]; (3) Aged 18–65 years; (4) Received chest computerized tomography (CT) imaging examination; (5) Co-existing pulmonary infections; (6) The requisite case records were complete and traceable.

Confirmation of TB was based on positive sputum or bronchoscopic lavage fluid smear results and/or positive culture results, and positive clinical response to anti-tuberculosis treatment; the diagnostic criteria for non-TB pulmonary infectious diseases (e.g., viral, fungal, bacterial pneumonia) were based on negative sputum or bronchoscopic lavage fast-acid smear results, negative MTB culture results, positive results of the corresponding pathogenic organism, and having no evidence for TB infection for at least one year after discharge from hospital.

Firstly, we retrospectively collected and collated the demographic and clinical data, and chest CT scan results of HIV-infected patients with confirmed TB co-infection (HIV/TB patients) and HIV-infected patients with pulmonary infections caused by pathogens other than MTB (HIV/non-TB patients). These were the first data collected when the patients were admitted to hospital. Weight loss was defined as no less than 5% weight loss from positive symptoms to admission [7]. Highest body temperature was that detected during the hospitalization period. Pulmonary cavitation, pulmonary lesion, effusion, and lymphadenopathy were identified on CT imaging. Lymphadenopathy was defined as the short axis diameter of a lymph node exceeding 10 mm on CT examination [8, 9].

The demographic, clinical, and chest CT data of these patients were compared by univariate data analysis. Mathematical differences in risk variables between the two groups of patients were deemed to be statistically significant if the calculated *p* value was found to be < 0.05. Subsequently, the significant continuous variables in the univariate analysis were discretized, and were assigned discrete values for different weighted levels of a particular variable.

Subsequently, we conducted a multivariate logistic regression analysis of all significant variables obtained via univariate analysis, including categorical variables and discretized variables, further identifying the variables that were significantly different between the two groups of patients. These significant variables after multivariate analysis were incorporated in the scoring system.

Moreover, during the course of multivariate logistic regression analysis, a regression coefficient was generated for these variables. A score would be assigned for each eligible variable according to its regression coefficient. The variable with the lowest regression coefficient was assigned a score of 1. Scores of other variables were linearly calculated (in units of 0.5) according to their regression coefficient ratios relative to that of the variable assigned a score of 1. The sum of the scores of all the variables indicates the likelihood of TB. Finally, a receiver operating characteristic (ROC) curve was plotted to decide the optimal cut-off scores that would distinguish TB and non-TB pulmonary pathology, depending on specificity and sensitivity.

**Fig. 1:**
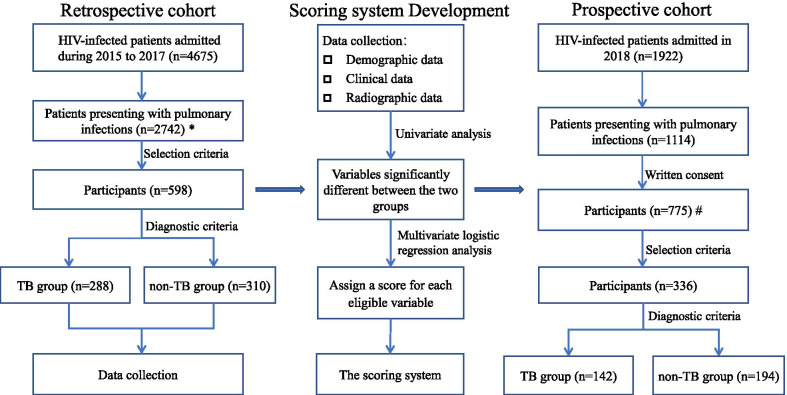
Flow chart of the study. *For 2742 cases presenting with pulmonary infections in the retrospective cohort, 2144 patients did not meet the selection criteria. Among them, 488 patients were excluded as they were younger than 18 years or older than 65 years of age. The remaining 1656 patients were excluded as requisite data were missing, among which were 98 for CT, 620 for ESR, 593 for PCT, 68 for CD4 + T-cell count, 193 for HIV RNA, 169 for ALB, and 138 for microorganism evidence. #For 755 cases in the prospective cohort, 419 patients were excluded as requisite data were missing, among which were 13 for CT, 145 for ESR, 143 for PCT, 18 for CD4 + T-cell count, 43 or HIV RNA, 44 for ALB, and 32 for microorganism evidence

Subsequent to the scoring system being established, we tested its accuracy in the retrospective cohort, as well as in a prospective cohort presenting with HIV infection and co-existing pulmonary infections. Written informed consent was required for each participant in the prospective cohort. Other selection criteria for the prospective cohort were the same as that of participants in the retrospective cohort. The study flowchart is illustrated in Fig. 1.

### Statistical analysis

Statistical data analysis was performed using Statistical Package for the Social Sciences (SPSS) 23.0 software (IBM, Armonk, NY). Differences between HIV/TB patients and HIV/non-TB patients were analyzed using the Mann–Whitney U test for numerical variables and the Chi-square test for categorical variables, with a value of *p* < 0.05 being considered statistically significant. Backward logistic regression analysis was utilized to further select variables that were significantly different (*p* < 0.05) between the two groups of patients, to obtain a regression coefficient, and assign a score for each variable.

## Results

### Patients and diagnoses

As shown in Fig. 1 and Table 1, 598 patients were included in our retrospective analysis, of which 288 were HIV-infected patients with confirmed TB co-infection (HIV/TB patients) and 310 were HIV-infected patients with non-TB pulmonary infections (HIV/non-TB patients). Most TB case confirmation (244/288) was based on combined positive culture and positive smear results, 6 cases were based on positive culture results only, and the diagnostic status of the remaining 38 cases was based on their positive smear results only. Moreover, all 288 patients exhibited a positive response to diagnostic anti-tuberculosis treatment. Among the non-TB group, fungal infection (52.58%) was the most common infection, followed by bacterial infection (40.00%), and viral infection (7.42%). When admitted, only a small proportion of subjects were receiving antiretroviral therapy (ART) (14.60% in the TB group and 19.70% in the non-TB group). Most subjects were highly immunodeficient (CD4 + T-cell count < 200 cells/μL), specifically 92.71% (267/288) in the TB group and 88.39% (274/310) in the non-TB group. All patients were admitted to the Division of Infectious Diseases of the Chongqing Public Health Medical Center between January 2015 and December 2017.

**Table 1:** Comparison of variables between HIV/TB patients and HIV/non-TB patients by univariate analysis

Variables	HIV/TB patients (n = 288)	HIV/non-TB patients				Z/F	*p*
		Viral infection (n = 23)	Fungal infection (n = 163)	Bacterial infection (n = 124)	Total (n = 310)		
Demographic							
Age (years)	43.73 ± 13.43	46.17 ± 8.98	45.55 ± 14.88	48.92 ± 14.04	46.94 ± 14.24	2.897	0.005
Male (n, %)	238(82.6%)	18(78.0%)	125 (76.7%)	96 (77%)	239 (77.1%)	2.841	0.092
Laboratory							
WBC (10^9^ cells/L)	6.11 ± 3.27	4.80 ± 1.77	5.55 ± 5.07	6.23 ± 3.59	5.77 ± 4.35	1.074	0.283
HGB (g/L)	98.66 ± 20.76	117.78 ± 24.94	113.31 ± 21.94	110.67 ± 25.63	112.59 ± 23.70	7.618	0.000
PLT (10^9^ cells/L)	201.16 ± 107.07	176.39 ± 110.85	218.09 ± 103.28	196.85 ± 90.75	206.50 ± 99.60	0.631	0.528
ALB (g/L)	32.54 ± 6.01	39.39 ± 5.20	34.83 ± 6.86	34.87 ± 6.46	35.19 ± 6.68	5.082	0.000
CD4^+^ T-cell count (cells/μl)	45 (1–591)	86 (6–669)	25 (1–504)	54.5 (2–1252)	37 (1–1252)	1.324	0.186
< 200	267(92.7%)	16(69.6%)	155(95.1%)	103(83.1%)	274(88.4%)	3.233	0.094
≥ 200	21(7.3%)	7(30.4%)	8(4.9%)	21(16.9%)	36(11.6%)		
HIV RNA (log10 copies/mL)	5.00 ± 1.43	3.89 ± 1.71	4.99 ± 1.15	4.26 ± 1.75	4.62 ± 1.51	1.900	0.257
ESR (mm/h)	75.31 + 42.62	51.50 ± 40.81	70.39 ± 34.80	58.69 ± 39.93	48.42 + 42.86	7.681	0.000
PCT (ng/ml)	0.15(0–73.99)	0.12(0–0.3)	0.14 (0.01–18.62)	0.12 (0.01–2.98)	0.09(0–18.62)	5.876	0.008
Symptoms							
Weight loss (Kg)	3(0–25)	2(0–10)	3(0–20)	3(0–20)	3(0–20)	1.689	0.091
Fever (n, %)	225(78.1%)	12 (52.2%)	94 (57.7)	74 (60%)	180 (58.1%)	27.487	0.000
Highest body temperature (°C)	38.79 ± 1.29	37.37 ± 0.95	37.89 ± 1.15	37.92 ± 1.12	37.86 ± 1.13	9.400	0.000
CT scan							
Number of infected pulmonary lobes (n)	5(0–5)	2 (0–5)	2 (0–5)	3 (0–5)	3 (0–5)	8.665	0.000
Opacities (n, %)	250(86.8%)	22 (95.7%)	128 (78.5%)	106 (85%)	256 (82.6%)	2.047	0.152
Solid lesion (n, %)	126(43.8%)	6 (26.1%)	43 (26%)	57 (46%)	106 (34.2%)	5.742	0.017
Pulmonary cavitation (n, %)	36(12.5%)	0 (0%)	4 (2%)	5 (4%)	9 (2.9%)	19.759	0.000
Pleural effusion (n, %)	150(52.1%)	0 (0%)	29 (18%)	11 (9%)	40 (12.9%)	105.724	0.000
Cervical lymphadenopathy (n, %)	33(11.5%)	1 (4.3%)	5 (3%)	4 (3%)	10 (3.2%)	15.162	0.000
Axillary lymphadenopathy (n, %)	11(3.8%)	3 (13.0%)	2 (1.2%)	1 (0.8%)	6 (1.9%)	1.919	0.166
Inguinal lymphadenopathy (n, %)	10(3.5%)	0	5 (3.1%)	1 (0.8%)	6 (1.9%)	1.354	0.245
Hilar and/or mediastinal lymphadenopathy (n, %)	212(73.6%)	1 (4.3%)	47 (29%)	44 (35%)	92 (29.7%)	115.3	0.000
Pericardial effusion (n, %)	37(12.8%)	2 (8.6%)	8 (5%)	3 (2%)	13 (4.2%)	14.592	0.000
Miliary nodules (n, %)	76(26.4%)	0 (0%)	2 (1%)	0 (0%)	2 (0.6%)	87.237	0.000

*WBC* white blood cell, *HGB* hemoglobin, *PLT* platelet, *ALB* albumin, *ESR* erythrocyte sedimentation rate, *PCT* procalcitonin

### Screening of potentially eligible variables for further analysis

We compared the mathematical differences of 24 risk variables between the two groups, using the Mann–Whitney *U* test for numerical variables and the Chi-square test for categorical variables. To avoid excluding potential variables that may differ between the two groups, we considered a *p* value of < 0.05 as being statistically significant, and included all variables with a *p* value of < 0.05 for further analysis.

As shown in Table 1, our univariate data analysis showed that the differences among 15 variables between HIV/TB patients and HIV/non-TB patients were statistically significant (*p* < 0.05), whereas 9 variables were not. The 15 statistically significant variables included age, hemoglobin, albumin, erythrocyte sedimentation rate (ESR), procalcitonin (PCT), fever, highest body temperature, number of infected pulmonary lobes, solid lesion, pulmonary cavitation, pulmonary effusion, cervical lymphadenopathy, hilar and/or mediastinal lymphadenopathy, pericardial effusion, and miliary nodules.

### Discretization of numerical variables

Among the 15 variables with a *p* value of < 0.05, there were seven numerical variables which needed to be discretized for further analysis. The discretization of the seven numerical variables is shown in Table 2. Each of the numerical variables was assigned an integer value of 0 to 4 based on their individual discretized ranges.

**Table 2:** Discretization of numerical variables

	0	1	2	3	4
Highest body temperature*(℃)	< 37.3	37.4–38.4	38.5–38.9	39.0–39.9	≥ 40.0
ESR*(mm/h)	< 20.0	20.1–49.9	50.0–79.9	80.0–99.9	≥ 100.0
ALB* (g/L)	≤ 30.0	30.1–32.9	33.0–35.9	36.0–39.9	≥ 40.0
HGB* (g/L)	< 60.0	60.1–89.9	90.0–109.9	110.0–119.9	≥ 120.0
PC * (%)	≤ 0.10	0.11–0.19	0.20–0.39	0.40–0.89	≥ 0.90
Number of infected pulmonary lobes # (n)	1	2	3	4	5
Age^#^ (y)	≤ 29	30–39	40–49	50–59	≥ 60

*HGB* hemoglobin, *ALB* albumin, *ESR* erythrocyte sedimentation rate, *PCT* procalcitonin

*Ranges are assigned according to the degrees of the abnormality in the clinical practice; ^#^Assigned by equal intervals

### Multivariate logistic regression analysis and establishment of the scoring model

Based on the seven discretized variables and the other eight significantly different categorical variables, multivariate logistic regression analysis was carried out to further identify the differences between the two groups. Among these 15 variables, eight variables were observed to be significantly different (*p* < 0.05, Table 3), suggesting that these parameters could be applied to distinguish TB from non-TB infections in HIV-infected patients.

**Table 3:** Backward logistic regression analysis of 15 variables and score assignment

Variables	Coefficient	*p*	Score
Fever	1.302	0.002	3.5
Highest body temperature	0.919	0.000	2.5
ESR	0.388	0.000	1
Cervical lymphadenopathy	1.269	0.009	3.5
Hilar and/or mediastinum lymphadenopathy	1.376	0.000	3.5
Pulmonary cavity	1.995	0.000	5
Pleural effusion	1.397	0.000	3.5
Miliary nodules	3.660	0.000	9.5
Age	1.597	.206	0
HGB	3.061	.080	0
ALB	1.029	.310	0
PCT	.055	.814	0
Number of infected pulmonary lobes	2.392	.122	0
Solid lesion	.056	.813	0
Pericardial effusion	.491	.484	0

*HGB* hemoglobin, *ALB* albumin, *ESR* erythrocyte sedimentation rate, *PCT* procalcitonin

These eight variables were therefore incorporated into the scoring model, in which, ESR was assigned a score of 1, as its regression coefficient was the lowest. Scores of other seven variables were linearly calculated (in units of 0.5) according to their regression coefficient ratios relative to that of ESR. The score assignment for each variable is shown in Table 3. The sum of the scores of each of the eight variables results in a total score, which represents the likelihood of TB, with a higher score indicating a higher probability of MTB infection.

### Model assessment in the retrospective cohort

**Fig. 2:**
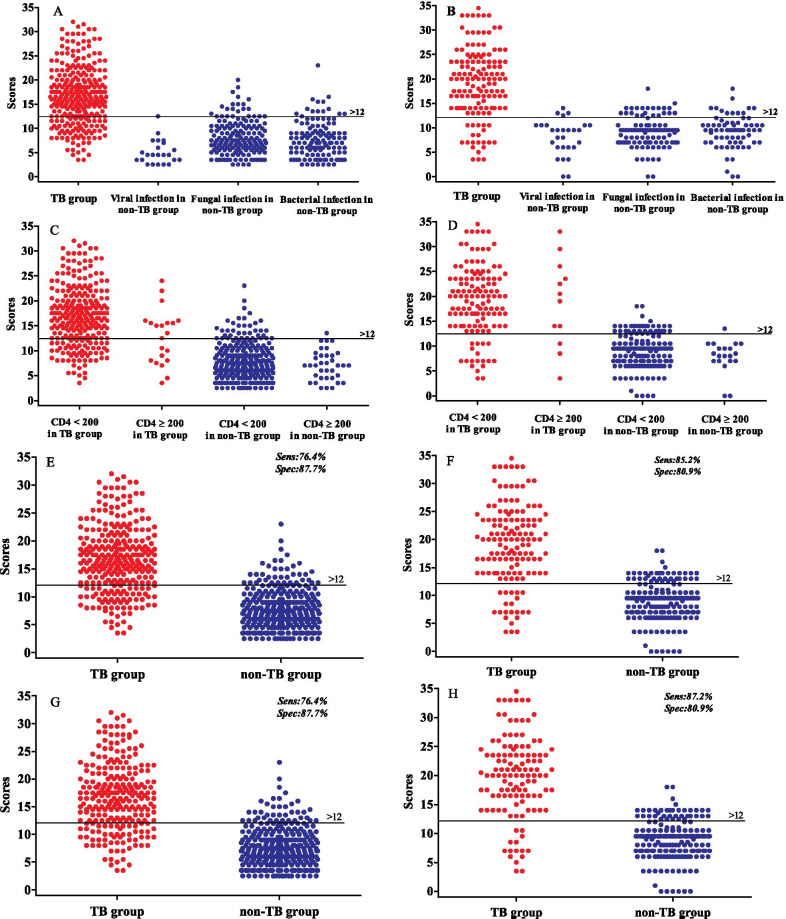
The score distribution of HIV-infected patients with TB and non-TB pulmonary infection in the retrospective and prospective cohorts. **A**, **C** and **E**: Retrospective cohort (n = 598); **B**, **D** and **F**: Prospective cohort (n = 336); **G** and **H**: Retrospective cohort (n = 560) and prospective cohort (n = 319), 38 cases and 17 cases respectively who were diagnosed as TB by smear-positivity and clinical response after anti-TB treatment, were excluded

**Fig. 3:**
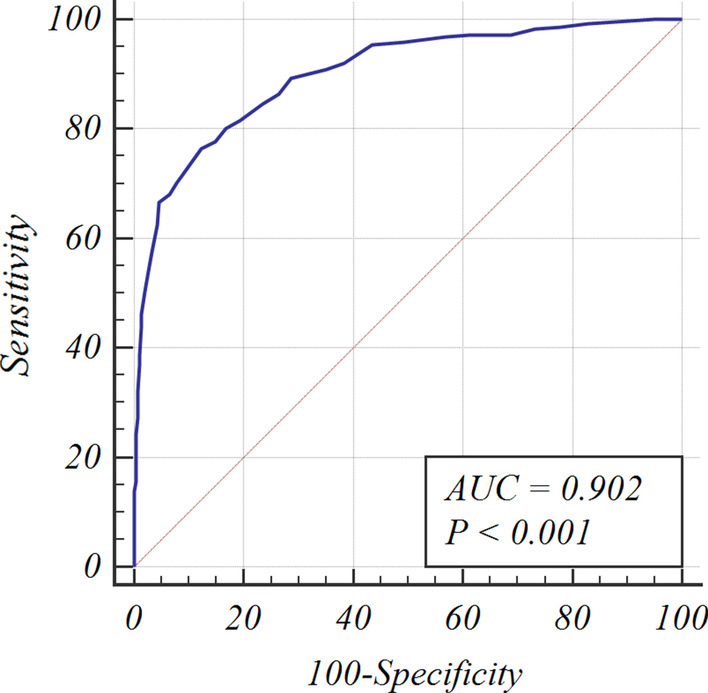
Receiver operator characteristic (ROC) curve for the scoring system. Each data point on the ROC curve represents a sensitivity/specificity pair corresponding to a particular decision threshold. The area under the ROC curve is a measure of how well a parameter can distinguish between two groups (TB/non-TB). *AUC* area under the ROC curve

The score distribution of 598 HIV-infected patients with TB or non-TB pulmonary infection in this retrospective cohort ranged between 2.5 and 32 (Fig. 2A, C and E). The total score of the TB group was 16.64 ± 6.00, significantly higher than that of the non-TB group (7.74 ± 3.59, *p* < 0.01). As shown in the Fig. 3, the specificity increased while sensitivity decreased gradually, when decision threshold increased, with the AUC of the ROC curve being 0.902 (*p* < 0.001, Fig. 3). We chose a total score greater than 12 as the optimal cut-off score. When this cut-off value was applied to the data from the cohort of 598 HIV-infected patients, the sensitivity and specificity of our scoring model was 76.4% (95% CI, 70.9%-81.1%) and 87.7% (95% CI, 83.4%-91.1%), respectively, and its Youden index {(sensitivity + specificity)-100}, which measures the performance of our scoring system, was 64.13%, indicating that our novel scoring model is effective for differentiating TB from non-TB pulmonary infection in HIV-infected patients.

**Fig. 4:**
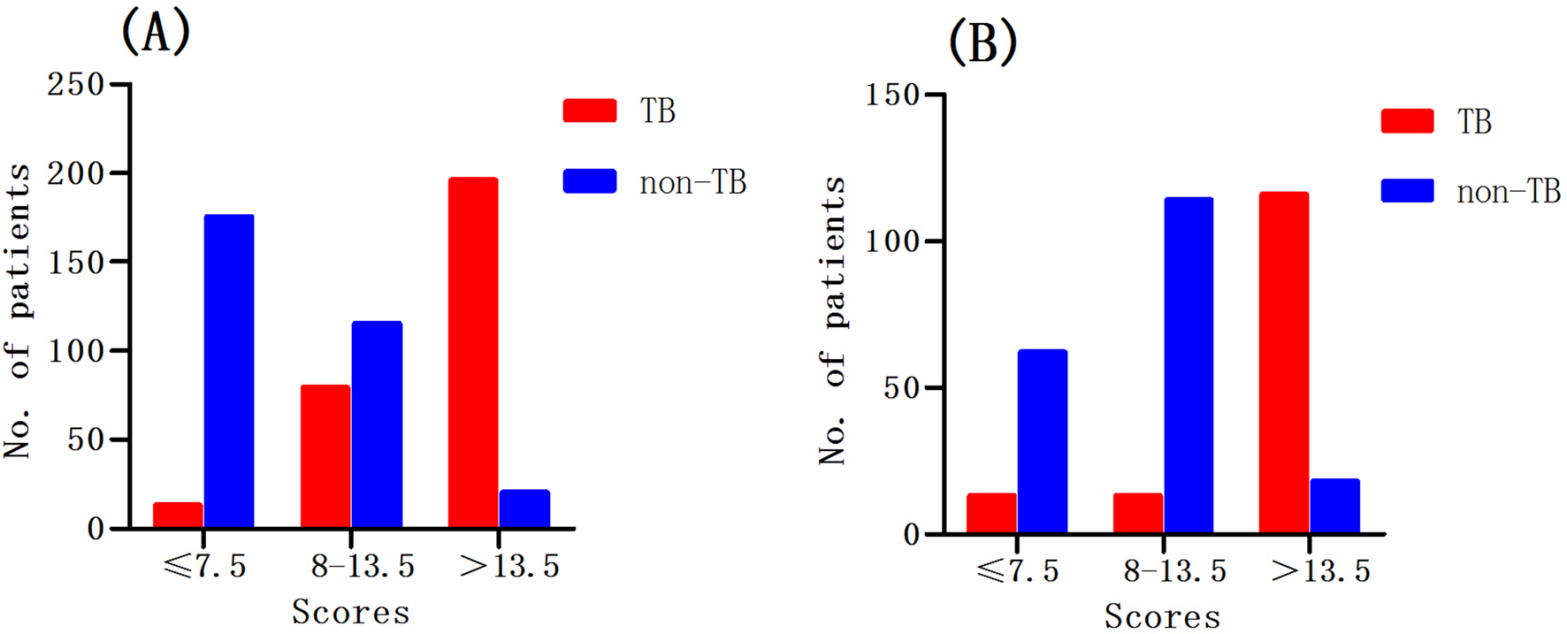
Number of patients in the three groups (TB unlikely, suspected TB, highly-suspected TB). TB unlikely: scores ≤ 7.5; suspected TB: scores 8–13.5; highly-suspected TB: scores > 13.5. **A** Retrospective cohort (n = 598); **B** Prospective cohort (n = 336)

Furthermore, to facilitate the application of this scoring model in a clinical context, we stratified 598 HIV-infected patients into three groups (“TB unlikely”, “suspected TB”, “highly-suspected TB”), depending on their respective likelihood ratios (Table 4). “TB unlikely” was defined by patient scores ≤ 7.5, where the corresponding negative likelihood ratios were below 0.1 (0.08). As shown in Fig. 4A, there were 188 patients whose scores were ≤ 7.5 in the retrospective cohort, 175 (93.1%) of which were confirmed as non-TB patients. On the other hand, “highly-suspected” TB was defined by patient scores > 13.5, where the corresponding positive likelihood ratios were above 10 (10.55). There were 216 “highly-suspected TB” cases in the retrospective cohort, 196 (90.7%) of which were proven to be TB patients (Fig. 4A). However, when the score was located in the range between 8.0 and 13.5, the category of patients proved difficult to define using our scoring model, and these patients were defined as “suspected TB”.

**Table 4:** Parameters of the scoring model using different cut-off scores as decision thresholds

Decision threshold	Sensitivity	Specificity	Positive likelihood ratios	Negative likelihood ratios
> 4.0	99.30	17.10	1.20	0.04
> 4.5	98.60	22.26	1.27	0.06
> 5.5	97.20	31.29	1.41	0.09
> 6.5	96.90	43.23	1.71	0.07
> 7.0	95.80	50.65	1.94	0.08
> 7.5	95.50	56.45	2.19	0.08
> 8.0	92.00	61.61	2.40	0.13
> 8.5	91.00	64.84	2.59	0.14
> 9.0	89.20	71.29	3.11	0.15
> 9.5	86.50	73.55	3.27	0.18
> 10.0	84.70	76.45	3.60	0.20
> 10.5	81.60	80.65	4.22	0.23
> 11.0	80.20	83.23	4.78	0.24
> 11.5	77.80	85.16	5.24	0.26
> 12.0	76.40	87.74	6.23	0.27
> 12.5	71.90	90.97	7.96	0.31
> 13.0	70.10	92.26	9.06	0.32
> 13.5	68.10	93.55	10.55	0.34
> 14.0	66.70	95.48	14.76	0.35
> 14.5	62.50	95.81	14.90	0.39
> 15.0	57.60	96.77	17.87	0.44

When each group was stratified according to CD4^+^ T-cell count, it was found that cavitation was significantly less common in those HIV-infected persons with CD4^+^ T-cell counts < 200 cells/μL than those with CD4^+^ T-cell counts ≥ 200 cells/μl, in both the TB group (10.86 vs 33.33%) and the non-TB group (2.55 vs 5.56%). For those with CD4^+^ T-cell counts < 200 cells/μl, as shown in Fig. 2C, the total score of the TB group was 16.94 ± 5.94, which is significantly higher than that of the non-TB group (7.84 ± 3.65, *p* < 0.01). The sensitivity and specificity of our scoring model for this specific subgroup was 77.90 and 86.5% respectively. For those with CD4^+^ T-cell counts ≥ 200 cells/μl, the total score of the TB group was 12.79 ± 5.37, which is also significantly higher than that of the non-TB group (7.03 ± 2.93, *p* < 0.01). The sensitivity and specificity of the scoring model in this subgroup was 57.14% and 97.22%.

It is worth noting that there were seven cases of non-tuberculous mycobacteria (NTM) in our cohort, and they were all identified as non-TB cases according to our scoring system, as their scores were not greater than 12. Moreover, once the 38 cases who were diagnosed as TB by smear-positivity and clinical response after anti-TB treatment were excluded, 76.40% of TB participants were identified as TB cases according to our scoring system (Fig. 2G), indicating that whether the 38 cases were excluded or not, the sensitivities were virtually identical {76.40% (191/250) vs 76.39% (220/288)}.

### Model Assessment in the prospective cohort

In order to establish the validity of the scoring model, 336 HIV-infected patients were included in a prospective cohort, of which 142 were confirmed as TB co-infection (HIV/TB patients) and 194 were non-TB pulmonary infections (HIV/non-TB patients). The majority of TB cases confirmation (125/142) was based on combined positive culture and positive smear results, and that of the remaining 17 cases was based on positive smear results only. All 142 patients had a positive response to diagnostic anti-tuberculosis treatment. In this prospective cohort, 47.89% in TB group and 30.41% in the non-TB group were treated with ART when they were admitted. Regarding CD4^+^ T-cell counts, 91.55% of cases (130/142) in the TB group and 89.69% of cases (174/194) in the non-TB group were highly immunodeficient.

Similar to the retrospective cohort, the total risk score in the TB group was significantly higher than that of the non-TB group (Fig. 2B, D, F and H, *p* < 0.01). When a total score of > 12 was used as the identifying criterion, the sensitivity and specificity of our scoring model was 85.2% and 80.9%, respectively, and its Youden index was 66.10%, further indicating that our scoring model is indeed effective for differentiating TB from non-TB pulmonary infection in HIV-infected patients.

Furthermore, these 336 patients were divided into a “TB unlikely” group, a “suspected TB” group, and a “highly-suspected” TB group. In the “suspected TB” group (7.5 < score ≤ 13.5), it remained difficult to identify TB from non-TB infections using our scoring model. However, our scoring system has been validated, with high predictive accuracy in the “TB unlikely” group and the “highly-suspected TB” group. There were 75 patients in the “TB unlikely” group (scores ≤ 7.5), of which 62 (82.7%) were confirmed as non-TB patients (Fig. 4B). Of the 134 patients in the “highly-suspected TB” group whose scores were > 13.5, 116 (86.6%) patients were confirmed as TB patients (Fig. 4B).

In this prospective cohort, cavitations were also associated with CD4^+^ T-cell counts. Compared to those HIV-infected individuals with CD4^+^ T-cell counts < 200 cells/μl, those with CD4^+^ T-cell counts ≥ 200 cells/μl were more likely to present with cavitations, either in the TB group (41.67 vs 13.80%) or the non-TB group (5.00% vs 4.60%). For the those with CD4 + T-cell counts < 200 cells/μl, the total score of the TB group was 19.39 ± 7.13, which was significantly higher than that of the non-TB group (9.16 ± 3.32, *p* < 0.01). The sensitivity and specificity was 86.2% and 79.3%, respectively, when the scoring model was applied in this subclass. Regarding those with CD4^+^ T-cell counts ≥ 200 cells/μl, the total score of the TB group was 18.71 ± 8.47, which was also significantly higher than that of the non-TB group (7.95 ± 3.15, *p* < 0.01). The sensitivity and specificity of the scoring model was 75.0% and 95.0%, respectively, for this population.

## Discussion

This study aimed to establish a diagnostic scoring system to accurately differentiate a possible diagnosis of pulmonary TB from a possible diagnosis of non-TB infection in HIV-infected patients. Although Xpert®MTB/RIF and sputum smear in concentrated form can distinguish between MTB and other infections, these technologies are not available in many resource-limited regions, and our proposed scoring system is useful in such scenarios. Several risk variables with different weights were incorporated and used in this model. The scoring model has been validated, and shows high specificity and sensitivity in the retrospective cohort as well as in the prospective validation cohort. Moreover, it is possible that all variables utilized by the scoring model can be acquired on the first day of hospital admission, making our scoring model a rapid and accurate diagnostic method. This novel scoring system could be used as an auxiliary diagnostic pathway, and effectively circumvent the limitations of current diagnostic technologies in a clinical setting, especially so in resource-limited settings.

In clinic, culture of MTB is considered the current reference standard for TB diagnosis [10]. However, culture requires sophisticated laboratory infrastructure and trained personnel [11, 12]. Moreover, the time required for culture growth is tediously long (10–21 days), which may unduly delay "timeous" critical decisions concerning patient treatment, especially in HIV-infected patients whose immunity is already compromised [11]. Smear microscopy is an inexpensive and rapid test with high specificity for TB diagnosis, while its sensitivity is disappointingly low, ranging between 35 and 80% [12–14]. Beside these conventional laboratory diagnostic techniques, molecular diagnostic tests, including the loop-mediated amplification test (LAMP), the GeneXpert assay, and the urine lipoarabinomannan (LAM) lateral flow assay have also been developed, and are used in establishing a diagnosis of TB in HIV-infected patients [15–18]. However, these molecular tests also have some limitations, such as requiring high-quality samples or sophisticated infrastructure, and lacking sensitivity. A study by Yuan et al. [19] (reporting in a meta-analysis that included 10 studies, with1920 suspected TB specimens), indicated an 80.0% sensitivity and 96.0% specificity for LAMP in the diagnosis of pulmonary TB. However, in HIV-infected patients, a lower sensitivity of 65% (95%CI: 48–79%) was observed when using LAMP [20]. Furthermore, diagnostic therapy using anti-tuberculosis agents is time-consuming and has low sensitivity, as these agents, such as rifampicin and pyrazinamide, are antibiotics, and their antimicrobial spectrum extends beyond MTB. Thus, in addition to current diagnostic techniques, the establishment of a simple, rapid, accurate and sensitive clinical diagnostic method for the early diagnosis of TB in HIV patients has become a research priority.

Diagnostic scoring systems are generally used to diagnose diseases or conditions without specific, disease-defining symptoms or signs, such as autoimmune hepatitis (AIH), disseminated intravascular coagulation (DIC), and the estimation of bleeding risk [21–27]. For HIV-infected patients, it is more difficult to differentiate pulmonary TB from non-TB pulmonary infections. Jarvis et al. reported a case of HIV-associated pulmonary cryptococcosis who was misdiagnosed as smear-negative pulmonary tuberculosis, which unfortunately had fatal consequences [28]. Shi et al. reported 24 HIV-infected patients who were misdiagnosed with lung cancers, of which 19 patients (79.2%) had tuberculosis [29].

In our study, we have developed and validated a diagnostic scoring system whose sensitivity and specificity exceeded 75%, in order to differentiate pulmonary TB from non-TB pulmonary infections in the context of pre-existing HIV infection. In this model, the risk variable of “miliary nodules” is assigned the largest scoring weight, with a score of 9.5. Miliary nodules have been described as a typical radiological manifestation of disseminated TB. They can also occur in a wide variety of other conditions [30–33]. In South Korea, a TB endemic region, the most common differential diagnoses for radiological miliary nodules are miliary TB (54%) and miliary metastases of malignancies (26%) [34]. A study in the US, which has low TB endemicity, also revealed that TB is the most common etiology for radiological miliary nodules (28.3%), followed by sarcoidosis, silicosis, and extra-thoracic malignancies [35]. Our results included 78 patients who had miliary nodules, 76 of whom were confirmed to have TB, and two who were confirmed as having non-TB pulmonary infection. Thus, the identification of miliary nodules was recognized as an important diagnostic factor for TB in our model. Moreover, pulmonary cavitation is also a classic hallmark of TB, and they are frequently present in patients diagnosed with pulmonary TB [36, 37]. These cavities offer an ideal growth environment for MTB, as the cavitary wall can limit drug penetration, and the presence of cavitary lesions has been shown to be associated with longer times for sputum culture conversion, and higher rates of treatment failure and relapse [38–41]. It is worth noting that cavitation is presented with thinner wall and is less prevalent in HIV-infected individuals with TB, and the incidence has been reported to range between 16 and 48% in this cohort, whereas more than 50% of immunocompetent pulmonary TB patients present with cavities [42–46]. Nevertheless, pulmonary cavitation is a distinct feature of patients with TB infection. In the present study, the incidence of pulmonary cavities in HIV-infected patients with TB (ranging between 10.86 and 41.67%) was significantly higher than that in non-TB subgroups (ranging between 2.55 and 5.56%), and thus this variable was allocated the second largest weight in our scoring model, with a score of 5.0. Other than miliary nodules and pulmonary cavities, the other 6 TB-associated variables which were obviously statistically different between the TB group and the non-TB group, were incorporated in our model, including fever, highest body temperature, ESR, cervical lymphadenopathy, hilar and/or mediastinum lymphadenopathy, and pleural effusion.

Based on our data, we propose that, using our diagnostic scoring system, patients with a total score greater than 12 have a significantly higher probability to have TB, and clinicians should consider initiating anti-tuberculous therapeutic drug regimens to these patients even if smear or culture positivity has not been established, thereby not delaying treatment. Conversely, patients with a score of no more than 12 have a relatively lower likelihood of a diagnosis of pulmonary TB, suggesting that clinicians exercise due diligence in the quest for other potential pulmonary pathogens. In addition, our diagnostic scoring system may be combined with the results of the likelihood ratio calculations, which reflects both sensitivity and specificity. When the positive likelihood ratio is above 10 (scores > 13.5), the diagnosis of TB is highly reliable, and when the negative likelihood ratio is lower than 0.1 (scores ≤ 7.5), this indicates that the patient is more likely infected with a lung pathogen other than MTB.

We acknowledge that our study has limitations, as the scoring system is less efficacious when the scores of patients ranged between 7.5 and 13, and if a patient’s score lies within this range, clinicians should resort to other, more specific, diagnostic methods to establish a definitive diagnosis. In addition, as the establishment of the scoring system was based on a retrospective design, some bias might exist. Moreover, as the diagnosis of TB was based on microbiological evidence and the effectiveness of diagnostic anti-tuberculosis treatment, we nevertheless may not have completely eliminated all false-positive errors. Finally, all the study participants were infected by MTB or by another opportunistic pathogenic microorganism, and thus were admitted to our hospital. All of the participants were AIDS patients, thus CD4^+^ T-cell counts for these patients were expectedly low (1–1252 cells/μl) in this study. Collectively, this study suggests that our scoring system is a viable means to distinguish between TB and other infections in Chinese AIDS patients.

## Conclusions

In this study, we developed a rapid diagnostic scoring system which could be helpful in differentiating pulmonary TB from non-TB pulmonary infections in HIV-infected patients. This novel scoring system could be used as an auxiliary diagnostic pathway, and effectively circumvent the limitations of current diagnostic technologies in a clinical setting, especially in the absence of facilities for MTB culture or Xpert®MTB/RIF. It has been validated, and showed high specificity and sensitivity in the retrospective cohort as well as in the prospective validation cohort. However, the clinical implications of this scoring model should be assessed in larger cohorts, and should include HIV positive and negative participants from different age groups and ethnicities. In addition, further investigations should be conducted to differentiate and ascertain the causes of pulmonary infections in HIV-infected patients when the scoring system does not support a TB diagnosis.

## Data Availability

The datasets studied and/or analyzed during the present study is available from the corresponding author on reasonable request.

## Abbreviations

ART: Antiretroviral therapy
AUC: Area under the curve
AIH: Autoimmune hepatitis
DIC: Disseminated intravascular coagulation
ESR: Erythrocyte sedimentation rate
HIV: Human immunodeficiency virus
LAM: Lipoarabinomannan
LAMP: Loop-mediated amplification test
MTB: *Mycobacterium tuberculosis*
PCT: Procalcitonin
ROC: Receiver operating characteristic
TB: Tuberculosis
